# Interpretation the Hepatotoxicity Based on Pharmacokinetics Investigated Through Oral Administrated Different Extraction Parts of *Polygonum multiflorum* on Rats

**DOI:** 10.3389/fphar.2018.00505

**Published:** 2018-05-25

**Authors:** Miao Zhang, Longfei Lin, Hongmei Lin, Changhai Qu, Lei Yan, Jian Ni

**Affiliations:** ^1^School of Chinese Materia Medica, Beijing University of Chinese Medicine, Beijing, China; ^2^Institute of Chinese Materia Medica, China Academy of Chinese Medical Sciences, Beijing, China; ^3^Fengtai District Community Health Center, Beijing, China

**Keywords:** *Polygonum multiflorum*, hepatotoxicity, pharmacokinetics, LC-MS/MS, different extraction parts

## Abstract

The liver injury induced by *Polygonum multiflorum* (PM) used for clinical treatment has recently received widespread attention. This study aimed to determine the hepatotoxicity of PM through pharmacokinetics studies. The extract of PM was separated to isolate the anthraquinone fraction, the tannin and polysaccharide fraction, the hydroxystilbene fraction, and the combined anthraquinone fraction. A rapid LC-MS/MS method was developed and validated to simultaneously analyze 2,3,5,4′-tetrahydroxystilbene-2-*O*-β-glucoside (TSG), emodin-8-*O*-β-D-glucopyranoside (EDG), and emodin in rat plasma, and was applied to the pharmacokinetics (PK) studies. The hepatotoxicity of different extracted parts of PM was evaluated through the levels of alanine aminotransferase (ALT), aspartate aminotransferase (AST), alkaline phosphatase (ALP), total bilirubin (TBil), direct bilirubin (DBil), and indirect bilirubin (IBil) in rat serum. The results showed that liver injury occurred in all the treated groups and that the hepatotoxicity performance of the total extract was different from other groups. The pharmacokinetic studies showed that the C_max_, T_max_, AUC, t_1/2_, and MRT of the major compounds of different extracted parts were significantly different in rat plasma at same dosage. Emodin-*O*-hex-sulfate, tetrahydroxystilbene-*O*-(galloyl)-hex, emodin (original and generated through EDG deglycosylation), and other free anthraquinones might be responsible for the hepatotoxicity of PM *in vivo*. PM extracts produced inhibitory effects on drug metabolic enzymes, include CYP3A4, CYP2C19, CYP2E1, UGT1A1, etc. And these effects may be related to its hepatotoxicity and pharmacokinetic behavior different. This information on hepatotoxicity and the pharmacokinetic comparison may be useful to understand the toxicological effects of PM.

## Introduction

*Polygonum multiflorum* (PM) is the roots of *polygonaceae* plants *radix Polygoni multiflori*. PM has anti-cancer, anti-aging, and hepatoprotective effects; it can also reinforce kidney function and blacken hair (Zhang et al., 2004, 2012; Chen et al., 2011; Lin et al., 2015b; Pang et al., 2015). However, the reports on hepatotoxicity, especially acute injuries, which arise from the consumption of PM, occur worldwide (But et al., 1996; Mazzanti et al., 2004; Panis et al., 2005). Emodin has been reported as an important chemical constituent in PM that induces liver cell damage, and induced significant apoptosis in a time-dependent manner, as determined by the morphological changes in drug-treated cells (Zhang et al., 2010; Li et al., 2012).

The ingredients in PM were separated by type by using a process described in our previous studies (Lin L.F. et al., 2017), which produced the free anthraquinone fraction (the content mainly including free anthraquinone), tannins and polysaccharide fraction (the content mainly including tannins and polysaccharide), hydroxystilbene fraction (mainly composed of polyhydroxystilbene, such as 2,3,5,4’-tetrahydroxystilbene-2-*O*-β-glucoside, TSG), and the combined anthraquinone fraction (mainly composed of combined anthraquinones, such as emodin-8-*O*-β-D-glucopyranoside, EDG). The components of PM that cause hepatotoxicity are mainly present in the hydroxystilbene part, as confirmed by experiments *in vitro*. The toxic ingredient of PM may be associated with tetrahydroxystilbene-*O*-(galloyl)-hex and emodin-*O*-hex-sulfate, which was speculated through *in vitro* experiments (Lin L.F. et al., 2017). In this study, the hepatotoxic of these extracts was investigated to further validate the above results *in vivo*. The pharmacokinetics of these extracts were also investigated through a rapid, specific, and sensitive LC-MS/MS that was developed and validated for the simultaneous determination of TSG, EDG, and emodin in rat plasma after the oral administration of the different extracted fractions of PM. More importantly, a deeper understanding of the hepatotoxic effects of PM is obtained through the comparison of the pharmacokinetic parameters of the main components (TSG, EDG, and emodin) of different extractions of PM.

## Materials and Methods

### Materials

Methanol (HPLC-grade) was purchased from Fisher (United States). Cascada^TM^ IX-water Purification System (Pall Co., United States) was used to provide high purity water. The standards of emodin and 2,3,5,4′-tetrahydroxystilbene-2-*O*-β-glucoside were provided by Shanghai Standard Biotech Co., Ltd (Shanghai, China), Emodin-8-*O*-β-D-glucopyranoside was provided by Shanghai yuanye Bio-Technology Co., Ltd (Shanghai, China).

### The Different Parts of PM Preparation and Determination

There are two main methods for the treatment of disease by using PM in a clinical setting. One is as a prescription, which is extracted with water; the other is used as a Chinese patent drug. The extraction methods of different patent drugs are different; water extraction or 70% ethanol extraction is generally used in the preparation of the patent drug that contains PM. Therefore, PM was extracted by 70% ethanol and then extracted with water in this study. This method ensured that all components in PM were extracted completely.

**FIGURE 1:**
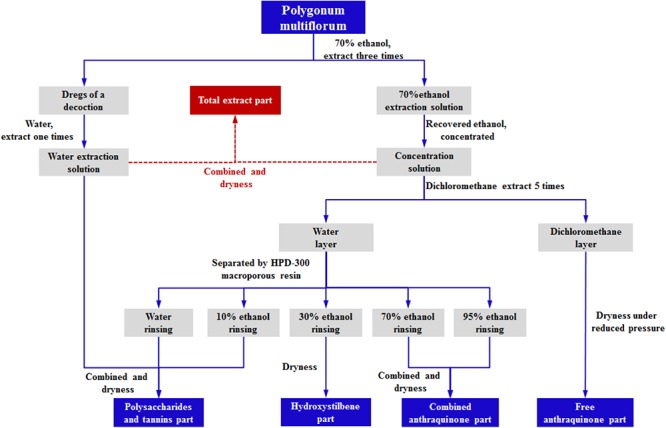
The process of the extraction of PM.

PM was decocted three times in 10-volumes of 70% ethanol and then decocted once with 10-volumes of water; each step lasted 1.5 h. The resulting filtrates were combined and concentrated to dryness under reduced pressure, which obtained the **“Total extract” (TE)**. Separately, PM was also decocted by the same methods, the 70% ethanol extracts were combined, the solution was extracted five times with the same volume of dichloromethane (DCM) after recovery and concentration, and then the DCM solution was combined and concentrated to dryness under reduced pressure, to obtain the **“Free anthraquinone fraction”** **(FAP)**. The fraction after extracted by DCM was separated by using HPD-300 macroporous resin, the water and 10% ethanol elution was combined to yield the **“Polysaccharides and tannins fraction” (PTP)**; the 30% ethanol elution produced the **“Hydroxystilbene fraction” (HDP)**; and the combination of the 70% ethanol and 95% ethanol elution was considered to represent the **“Combined anthraquinone fraction” (CAP)** (Lin L.F. et al., 2017). The process of the extraction is shown in **Figure 1**. The analysis of emodin, TSG, and EDG in the different PM fractions was performed by using a Prominence LC-20A HPLC system equipped with a SPD-20A UV-Detector (SHIMADZU Corporation, Japan).

### Hepatotoxicity of Different Parts of PM

#### Animal Experiments

One-hundred and twenty male Sprague-Dawley rats (160 g ± 10 g) were used as supplied by Beijing Vital River Laboratory Animal Technology (Beijing, China). All rats were maintained in plastic cages under standard environmental conditions 26 °C ± 2 °C with a relative humidity of 50% ± 10% and 12-h dark/light cycle. The rats were fed on a standard rat diet and water *ad libitum*. Experiments were conducted after a 7 days acclimatization period. All experiments were performed during the daytime.

#### Dose and Group

The rats were randomly divided into six groups (20 animals/per group). In the sixth group (the control group), rats were administered an equal volume of blank solvent only. The rats in groups 1–5 were treated with different fractions of PM (Total extract, PTP, HDP, CAP, and FAP, respectively) at 6 g/kg (calculated on the quantity of crude material) of body weight per day. The dried extract was re-dissolved, dispersed in 0.5% CMC-Na solution, and then intragastrically administered to rats for 90 days; the weight and diet of the rats were recorded every day. After final administration, the rats were anesthestized through the intraperitoneal injection of chlorine hydrate. Subsequently, blood was sampled from the aorta abdominalis and the serum was separated and stored at -80°C for the measurement of the biochemical indexes.

#### Biochemical Analysis

Alanine aminotransferase (ALT), aspartate aminotransferase (AST), Alkaline phosphatase (ALP), total bilirubin (TBil), direct bilirubin (DBil), and indirect bilirubin (IBil) were quantitatively analyzed by using a Glamour 4000 biochemical analyzer (Glamour Co., Ltd., United States).

### Pharmacokinetic Study of Different PM Fractions

#### Instrumentation and Chromatography

The detection of TSG, EDG, and emodin was performed by using an Agilent 1290 (Agilent, United States) liquid chromatograph equipped with a QQQ (Agilent 6460, United States) tandem mass spectrometer. The UHPLC separation of all samples was performed on an ACQUITY UPLC HSS T3 C_18_ reversed-phase column (1.7 μm, 2.1 × 100 mm, Waters Co.) with a flow rate of 0.2 mL min^-1^. The auto-sampler was set at 4°C and the injection volume was 5 μL. The mobile phase consisted of acetonitrile solution (A) and 0.1% formic acid aqueous solution (B). The following gradient elution was used: 0–1 min, 20% A; 1–4 min, 20%–30% A; 4–6 min, 30%–100% A; 6–7 min, 100% A; 7–7.1min, 100%–20% A; 7.1–10 min, 20% A.

The mass spectrometer was operated in negative ion mode for the detection of TSG, EDG, emodin, and hyperin (the internal standard [I.S.]) with multiple reaction monitoring (MRM). The mass spectrometric parameters were as follows: gas temperature, 350°C; gas flow rate, 8 L min^-1^; nebulizer, 45 psi; capillary, 3500 V. Other mass spectrometric parameters were also optimized for the maximum sensitivity of each analyte, as shown in **Table 1**.

**Table 1:** Optimized multiple reaction monitoring parameters for the detection of analytes and internal standards (^∗^quantitative ion).

Compound	Precursor Ion(m/z)	Product Ion(m/z)	Fragmentor	CE
TSG	405	243^∗^	135	10
		143		60
EDG	431	269^∗^	180	20
		224		50
Emodin	269	225^∗^	135	20
		195		40
Hyperin (I.S.)	463	300^∗^	160	20
		271		40

#### Preparation of Calibration Standards and Quality Control (QC) Samples

The primary stock solutions of TSG, EDG, emodin, and the I.S. were prepared individually in methanol at a concentration of 0.1 mg mL^-1^, aliquoted, and stored at -20°C. The stock solutions was further diluted to concentrations between 10 ng mL^-1^ to 10 μg mL^-1^ (I.S. solution at 2 μg mL^-1^) to prepare working standard solutions with in acetonitrile-water (1: 4, v/v). The calibration standards were prepared by spiking control rat plasma with the working solutions of TSG, EDG and emodin to yield concentrations between 1 and 1000 ng mL^-1^. Low-, medium-, and high-concentration QC samples, which were used to determine the precision of the method, were prepared at 5, 50, and 500 ng mL^-1^ TSG, EDG and emodin in the same manner; the I.S. was 200 ng mL^-1^ for each sample.

#### Sample Preparation

A 50 μL aliquot of rat plasma and 5 μL of the I.S. was transferred to a 0.5 mL plastic tube and extracted by employing a liquid-liquid extraction technique measure. A 0.5 mL aliquot of ethyl acetate-acetone (10: 1) solution was added to the plasma sample and vortexed for 60 s. The plasma sample was then centrifuged at 14,000 rpm for 5 min to separate the supernatant. The extraction process was repeated and the two supernatants were combined. The supernatant was evaporated to dryness in a centrivap concentrator (Labconco, Kansas City, MO, United States) at 40°C. The residue was reconstituted in 50 μL of the mobile phase, vortexed mixed for 30 s, and centrifuged at 14,000 rpm for 5 min. The supernatant was removed and 5 μL was injected for LC-MS/MS analysis.

#### Method Validation

The method was developed and conducted according to the Bioanalytical Method Validation of U.S. Food and Drug Administration (FDA) guidelines with respect to specificity, linearity, the lower limit of quantification (LLOQ), accuracy, precision, sample dilution, recovery, matrix effects, and stability.

The selectivity of this method was investigated for the interference from endogenous components in samples, by a comparison of blank plasma from six different sources that contain analytes to the I.S. The linearity of the method was assessed through the assay of calibration curves in plasma between 1 and 1000 ng mL^-1^. The LLOQ (lower limit of quantification) was defined as the lowest concentration in the calibration curve with acceptable precision (RSD) of no more than 20% and accuracy within 100% ± 20%. The intra- and inter-day precision and accuracy were examined in six replicates of the QC samples analyzed on the same day and three consecutive days. The precisions were expressed as the RSD, which did not exceed 15%, and an accuracy < 15% of the nominal value. The extraction recovery and matrix effect were also determined by the assay of six replicates samples at three QC levels (six samples each), for which the RSD should be less than 15%. The stability of the analytes in plasma was investigated in the following conditions: 24 h storage at 4°C, three freeze-thaw cycles, and storage at -20°C for 1 month.

#### Pharmacokinetics

Thirty male Sprague-Dawley rats (250 g ± 10 g) were obtained from Vital River Laboratories (Beijing, P.R. China) and randomly divided into five groups. All rats were kept in an environmentally controlled breeding room and maintained on a 12 h dark/light cycle for at least 3 days before use. Food was withdrawn 12 h before administration of the test compound. The different fractions of PM (TE, FAP, PTP, HDP, and CAP) were orally administered to the five groups (six rats per group) at 6 g/kg of body weight, which was computed based on the quantity of crude material. Blood samples (0.3 mL) were collected from the ocular fundus veins of rats into heparinized microcentrifuge tubes before administration and at 10, 20, 30, and 45 min, and 1, 2, 4, 8, 12, and 24 h after oral administration. All blood samples were centrifuged at 10,000 rpm for 10 min to collect plasma and stored at -20°C until analysis. The pharmacokinetic parameters of TSG, EDG and emodin were computed by using Kinetica 4.4 software (Thermo Scientific, United States). Non-compartmental analysis was used to determine standard pharmacokinetic parameters of analytes. The data are presented as the mean ± SD.

### Western Blotting Assays of Hepatic Metabolic Enzymes

Thirty six male Sprague-Dawley rats (160 ± 10 g) were randomly assigned to six groups. Five fractions of PM (TE, PTP, HDP, CAP and FAP) were respectively mixed in 0.5% CMC-Na solution, and then were single given orally to mice. The dosage of the extract groups was at 6 g/kg of body weight (calculated on the quantity of crude material). The control group was administrated at an equal volume of blank solvent only. Liver samples were drawn from rats after 2 h of administration. Subsequently, liver samples were stored in the liquid nitrogen for the analysis of hepatic metabolic enzymes (CYP1A2, CYP2A, CYP2C9, CYP2C19, CYP2E1, CYP3A4, UGT1A1 and UGT1A8) by western blot.

Western blot is a commonly used method in molecular biology, biological chemistry and immunological genetics that can be used in qualitative and semi-quantitative analyses of protein. Coloring location and depth can be analyzed to evaluate the expression of protein in cells or tissues. In this study, proteins extracted from liver tissues of each group were used in western blotting to validate the pharmacokinetic results. The control group and the administrated group samples were used in western blotting analyses, each sample was repeated for three times. Thirty microgram of proteins from each sample were first resolved in the 10% SDS-PAGE gel and then transferred onto a nitrocellulose membrane (Bio-Rad, Hercules, CA, United States). The membrane was rinsed with PBS and the non-specific binding sites were blocked in a solution of 5% non-fat milk in PBST (0.05% Tween 20 in PBS) for 2 h at 37°C, followed by three times washes in PBST. The membrane was first incubated with CYP1A2, CYP2E1, CYP3A4 antibody (1:2000 dilution), CYP2A (1:500 dilution), CYP2C9, CYP2C19, UGT1A1 (1:1000 dilution) and UGT1A8 (1:3000 dilution) overnight and then washed in PBST buffer.

### Data Statistics and Ethics Statement

Statistical analysis was performed using an unpaired *T*-test. *P*-values lower than 0.05 were considered to be statistically significant. All data were expressed as the mean ± SD for all experiments.

The principles of laboratory animal care were followed and all procedures were conducted according to the guidelines established by the National Institutes of Health. Every effort was made to minimize animal suffering. This study was approved by the Animal Ethics Committee of the Beijing University of Chinese Medicine.

## Results

### Hepatotoxicity of Different PM Fractions

#### Body Weight and Dietary Intake Observation

**FIGURE 2:**
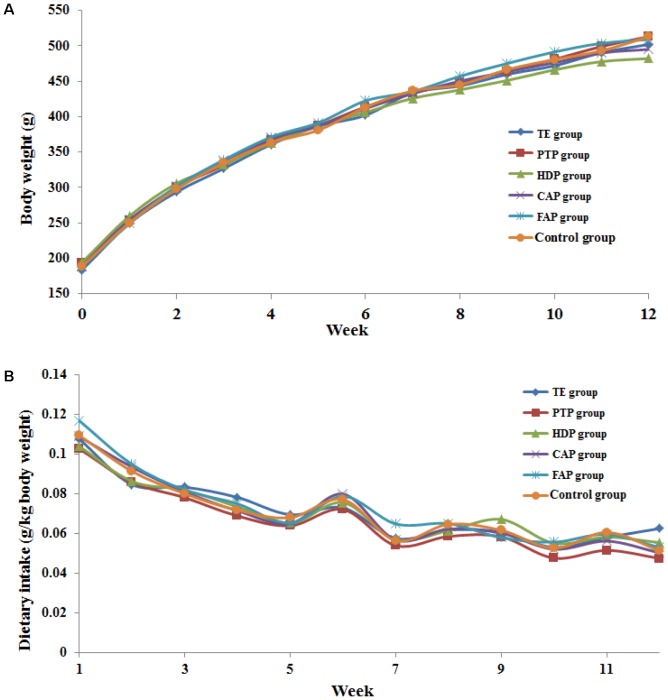
The increases of the body weight **(A)** and dietary intake **(B)** about 6 groups rats in 12 weeks.

The body weight and dietary intake of the six groups of rats that were administered the different fractions of PM and blank solvent were recorded for the 90 consecutive administration days. The dietary intake was calculated based on the rat body weight (expressed as g/kg body weight). The relative body weight and dietary intake of all the groups of rats were not significantly lower compared with the control group over 90 consecutive days (**Figure 2**). However, the increase in body weight of rats in the HDP group was lower than that of the other groups, which indicated that a toxic reaction may have occurred in this group of rats.

#### Changes of Biochemical Indexes Related to Hepatocyte Injury

**FIGURE 3:**
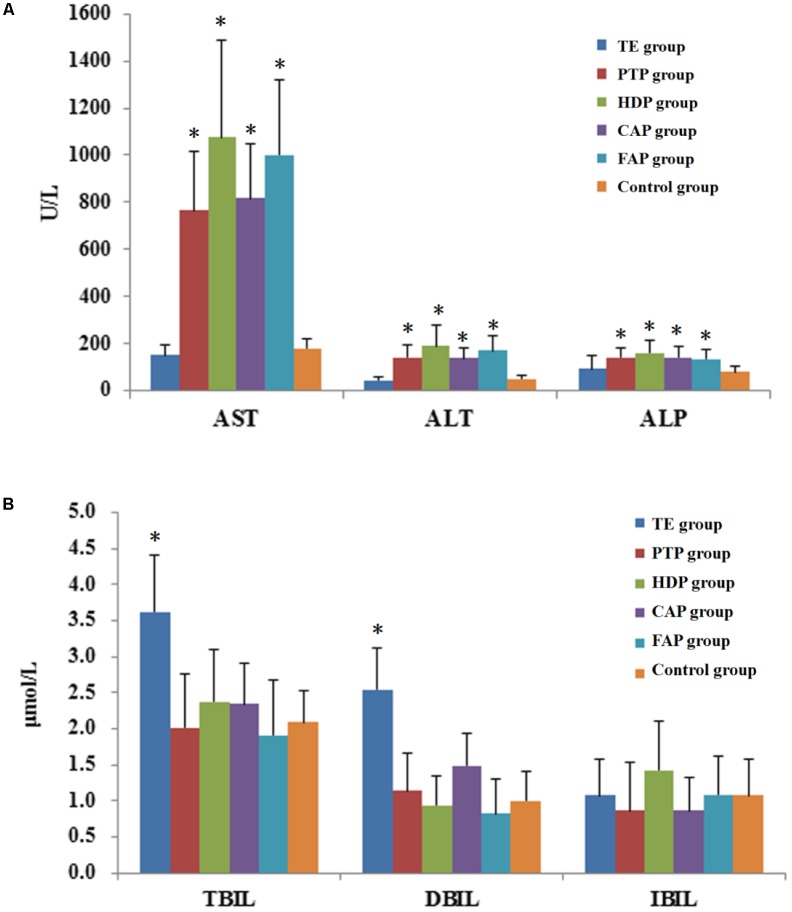
The biochemical indexes in rat plasma after oral administrated different parts of *Polygonum multiflorum.* **(A)**: The level of ALT, AST and ALP;**(B)**: The level of TBil, DBil, and IBil. **^∗^***P* < 0.05, comparison between the control group and treatment groups. (This data was published in Cell Physiol Biochem 2017;43:2102-2116).

ALT, AST, and ALP are biochemical indexes that could reflect the level of hepatocyte injury. ALT is the most sensitive index of liver function damage recommended by WHO; it is significantly increased in serum by only 1% hepatocellular necrosis. Serum ALT activity levels are the most frequently used laboratory indicator of hepatotoxicity. AST and ALT secretion reflect the specific function of liver cells. The results showed that the serum AST, ALT, and ALP levels in the PTP, HDP, CAP, and FAP groups were significantly higher than those of the control group. Among the different groups, the serum AST and ALT levels of HDP group were most obviously increased (**Figure 3A**). These results indicated the occurrence of liver injury in these four groups of rats. However, it should also be noted that the serum AST, ALT, and ALP levels of the TE group were not significantly higher than those of the control group.

#### Changes in Biochemical Indexes Related to Hepatic Secretory and Excretory Functions

The biochemical indicators for hepatic secretory and excretory functions include TBil, DBil, and IBil, which are all measurements of bilirubin. DBil is increased in serum if DBil cannot be normally converted into bile or if bile excretion is blocked. IBil is increased in the serum when the liver can not completely convert IBil to DBil owing to excessive erythrocyte destruction in the body. Hemolytic jaundice, hepatocellular jaundice, and obstructive jaundice can be diagnosed through the determination of the levels of TBil and DBil in serum. The results showed that the serum TBil and DBil levels of the TE group were significantly higher than those of the control group, but the other treatment groups did not exhibit this changed (**Figure 3B**). This indicated that the mechanism of liver injury in total extract-treated group was different from other groups, and may included bile duct blockage, jaundice hepatitis, and hemolytic disease.

The parenchymal liver injury was observed from the pathological liver tissue sections of the treatment groups, including cell swelling, ballooning of degenerating cells, and focal infiltration of inflammatory cells in the central vein (Lin L. et al., 2017).

### LC-MS/MS Method Validation

#### Specificity

**FIGURE 4:**
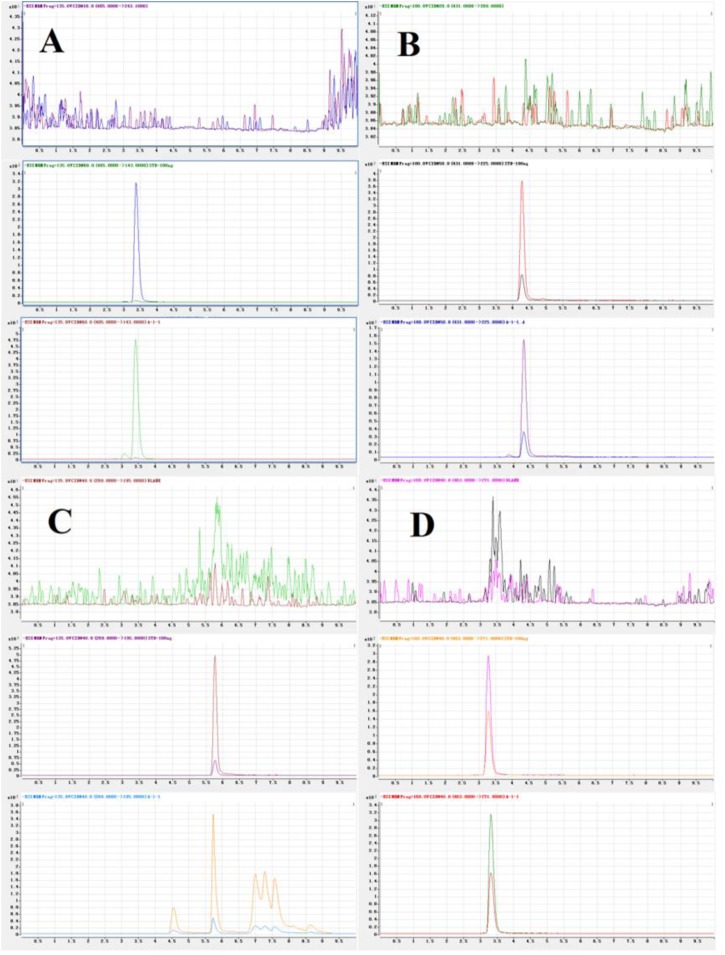
Representative MRM chromatograms of TSG **(A)**, EDG **(B)**, emodin **(C),** and I.S. **(D)**. (Up to down, Blank plasma; Blank plasma spiked three analytes and I.S., Plasma sample).

Typical chromatograms of the three analytes and the I.S. were presented in **Figure 4**. The retention times of TSG, EDG, emodin, and I.S. were 3.37, 4.27, 5.76, and 3.28 min, respectively. The results confirmed that this method was specific and that there was no significant interference in the blank plasma at the retention time of the analytes and the I.S. after analysis of the individual blank plasma samples from six different sources.

#### Linearity and LLOQ

The method presented good linearity and reproducibility over the concentration range of 1.0–1000 ng/mL, the correlation coefficient (r) was 0.99 or greater by weighted (1/x) linear regression analysis, and the range of each of these three compounds had at least six concentrations. The typical equations are shown in **Table 2**, where Y represents the peak area ratio and X represents the plasma concentration of analytes. The LLOQs for TSG, EDG, and emodin in rat plasma were 1.0 ng/mL with an RSD < 20%; the precision and accuracy were within the acceptable criteria.

**Table 2:** The calibration curves for TSG, EDG, and emodin.

Compound	Typical equations	*R*^2^	Weigh	Concentration range	LLOQ (RE%)
TSG	*Y* = 1.949 × X + 0.0457	0.999	1/X	1∼1000 ng	102.9 ± 15.2
EDG	*Y* = 2.032 × X + 0.1037	0.999	1/X	1∼1000 ng	105.9 ± 15.8
Emodin	*Y* = 2.204 × X + 0.0249	0.997	1/X	1∼1000 ng	89.19 ± 5.6

#### Precision and Accuracy

The intra- and inter-day precision and accuracy were investigated through the analysis of three QC levels (5, 100, and 1000 ng/mL) with six replicates samples on the same day and three consecutive validation days. The result showed that the inter- and intra-day precision of the method was within 15%, as summarized in **Table 3**. The intra- and inter-day RSD was between 2.11% and 11.86% and 2.49% to 10.74%, respectively. The results indicated the accuracy and precision of the method was acceptable.

**Table 3:** Accuracy and precision for the analysis of TSG, EDG and emodin in rat plasma.

Compound	Added concentration (ng/mL)	Intra-day (*n* = 6)			Inter-day (*n* = 18)		
		Found concentration (ng/mL) (mean ± SD)	Accuracy (%)	Precision (RSD%)	Found concentration (ng/mL) (mean ± SD)	Accuracy (%)	Precision (RSD%)
TSG	5	5.00 ± 0.47	100.09 ± 9.42	9.41	4.92 ± 0.46	98.36 ± 9.28	9.44
	50	47.82 ± 5.52	95.63 ± 11.04	11.55	48.76 ± 5.24	97.53 ± 10.47	10.74
	500	537.23 ± 11.32	107.45 ± 2.26	2.11	537.16 ± 13.39	107.43 ± 2.68	2.49
EDG	5	4.55 ± 0.54	91.04 ± 10.80	11.86	4.72 ± 0.43	94.41 ± 8.57	9.07
	50	50.89 ± 2.88	101.78 ± 5.77	5.67	51.77 ± 3.63	103.53 ± 7.27	7.02
	500	524.88 ± 31.69	104.98 ± 6.34	6.04	513.62 ± 28.26	102.72 ± 5.65	5.50
Emodin	5	5.58 ± 0.26	111.56 ± 5.29	4.74	5.35 ± 0.36	107.02 ± 7.12	6.65
	50	56.37 ± 2.12	112.74 ± 4.25	3.77	54.85 ± 2.60	109.69 ± 5.19	4.73
	500	511.90 ± 15.78	102.38 ± 3.16	3.08	510.11 ± 37.39	102.02 ± 7.48	7.33

#### Extraction Recovery and Matrix Effect

The extraction recovery and matrix effect results of TSG, EDG, and emodin are presented in **Table 4**. The precision of recoveries of TSG, EDG, and emodin between 6.71 to 13.37. For the matrix effect, the accuracy ratios of these analytes were in the between 89.68% and 102.17%, and no matrix components in the plasma indicated significant interference of the MS/MS responses of all analytes. These results indicated that the extraction recovery and matrix effects of this method met the FDA guidelines.

**Table 4:** Recoveries and matrix effects of TSG, EDG, and emodin.

Compound	Compound concentration(ng/mL)	Recovery (%)	Precision (RSD%)	Matrix effects (%)	Precision (RSD%)
TSG	5	92.80 ± 12.14	13.37	89.77 ± 4.36	4.85
	50	84.33 ± 8.12	9.63	94.01 ± 3.45	3.67
	500	76.98 ± 4.77	6.20	94.20 ± 4.35	4.62
EDG	5	107.85 ± 9.29	8.61	92.84 ± 4.38	4.74
	50	72.86 ± 7.90	10.84	91.01 ± 3.28	3.60
	500	67.36 ± 6.17	9.16	89.68 ± 3.64	4.06
Emodin	5	81.26 ± 5.45	6.71	101.79 ± 8.48	8.33
	50	78.21 ± 9.17	11.73	98.08 ± 6.08	6.20
	500	76.17 ± 7.86	10.32	102.17 ± 8.52	8.34

*The data are represented as the as the mean ± SD, *n* = 6*.

#### Stability

The stability of TSG, EDG, and emodin was investigated through the determination of the concentrations of three QC levels of plasma under the three tested conditions. All the values are shown in **Table 5**. Three concentrations of the three analytes in plasma were not significantly changed after storage at -20°C for 1 month, at 4°C for 24 h, and or three freeze-thaw cycles. These results showed that the three analytes were stable in the abovementioned, and that the established method was suitable for sample analysis.

**Table 5:** Stability of TSG, EDG, and emodin under various storage conditions.

Compound	concentration (ng/mL)	Long-term (-20°C)		Freeze–thaw		Short-term(24 h)	
		Found concentration (ng/mL)	Accuracy (%)	Found concentration (ng/mL)	Accuracy (%)	Found concentration (ng/mL)	Accuracy (%)
TSG	5	4.73 ± 0.57	94.62 ± 11.38	5.02 ± 0.35	100.36 ± 7.04	4.87 ± 0.23	97.30 ± 4.66
	50	50.21 ± 4.66	100.42 ± 9.32	48.26 ± 6.09	96.52 ± 12.19	48.60 ± 4.72	97.21 ± 9.45
	500	533.15 ± 14.76	106.63 ± 2.95	541.11 ± 15.01	108.22 ± 3.00	548.65 ± 8.12	109.73 ± 1.62
EDG	5	4.80 ± 0.46	96.01 ± 9.22	4.81 ± 0.26	96.18 ± 5.23	4.70 ± 0.49	94.10 ± 9.90
	50	51.79 ± 4.02	103.58 ± 8.04	52.62 ± 4.32	105.24 ± 8.64	53.22 ± 4.34	106.43 ± 8.67
	500	502.26 ± 13.10	100.45 ± 2.62	513.72 ± 35.11	102.74 ± 7.02	517.46 ± 28.98	103.49 ± 5.80
Emodin	5	5.26 ± 0.46	105.26 ± 9.19	5.21 ± 0.24	104.25 ± 4.70	5.22 ± 0.56	104.49 ± 11.14
	50	53.46 ± 2.91	106.92 ± 5.82	54.71 ± 2.20	109.43 ± 4.40	53.29 ± 3.91	106.57 ± 7.83
	500	506.47 ± 38.44	101.29 ± 7.69	511.95 ± 54.80	102.39 ± 10.96	525.17 ± 39.13	105.03 ± 7.83

*The data are represented as the mean ± SD, *n* = 6*.

### Pharmacokinetics Study

**FIGURE 5:**
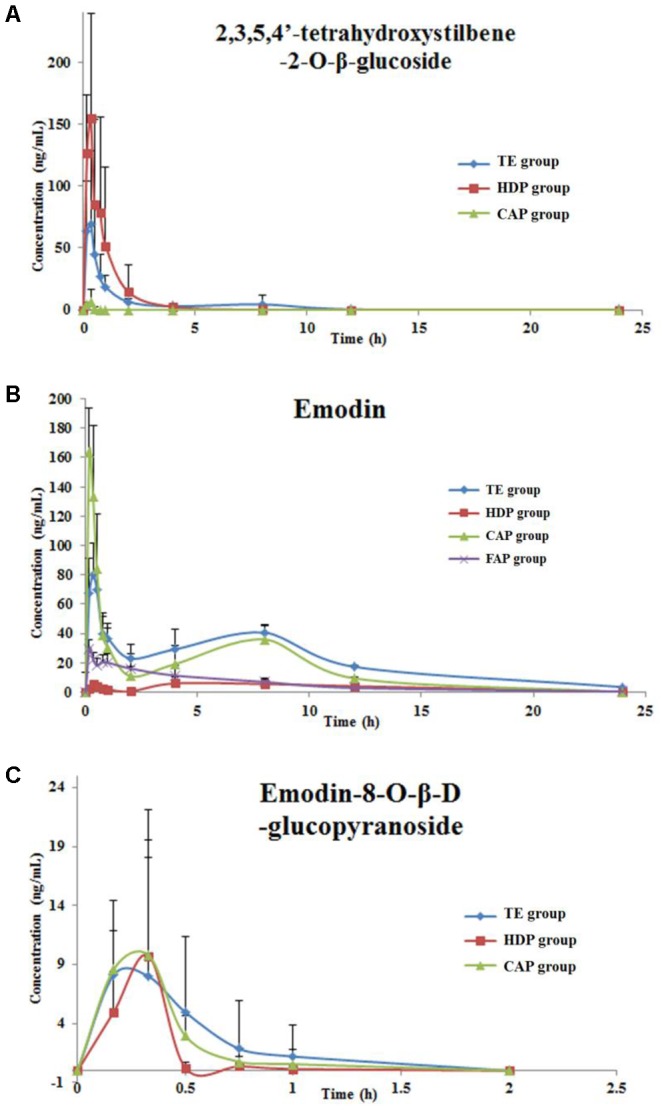
The mean (±SD) plasma concentration–time profiles of TSG **(A)**, emodin **(B)** and EDG **(C)** in rats after oral administration.

In the present study, LC-MS/MS method was successfully applied to quantify the drug in rat plasma obtained from five group rats that were given an orally administration of different parts of PM (TE, FAP, PTP, HDP, and CAP). The dosage of three compounds in different parts of PM administered for rats are summarized in **Table 6**. Because the content of the TSG, EDG, and emodin were lower in the PTP extract, these three components were not detected in rat plasma. The plasma concentration-time profiles and pharmacokinetic parameters of TSG, emodin and EDG of these groups are shown in **Figure 5** and **Table 7**.

**Table 6:** The dosage of the three compounds in different part of PM administered for rats.

Group	Emodin (mg/Kg)	TSG (mg/Kg)	EDG (mg/Kg)
TE group	4.91	212.23	22.04
PTP group	0.07	7.50	1.01
HDP group	0.11	156.35	3.93
CAP group	0.23	34.23	17.87
FAP group	3.67	0.17	0.08

**Table 7:** Pharmacokinetic parameters of TSG, emodin and EDG in rats.

Parameters	Unit	TSG			Emodin				EDG		
		TE group	HDP group	CAP group	TE group	HDP group	CAP group	FAP group	TE group	HDP group	CAP group
T_max_	h	0.25 ± 0.14	0.3 ± 0.07	0.28 ± 0.14	1.61 ± 3.13	4.81 ± 3.74	0.22 ± 0.08	1.11 ± 1.59	0.28 ± 0.13	0.25 ± 0.09	0.2 ± 0.07
C_max_	ng/mL	69.62 ± 51.79	211.39 ± 100.81^∗^	7.83 ± 8.04	86.66 ± 19.74	8.6 ± 2.2	165.06 ± 0.14^#^	30.96 ± 4.03^#^	21.52 ± 25.64	10.82 ± 7.73	17.91 ± 15.35
AUC_0-24_	ng h/mL	80.39 ± 34.36	134.08 ± 94.04	1.2 ± 0.92	480.03 ± 66.78	50.69 ± 9.5^∗^	314.48 ± 56.72^#^	121.43 ± 43.50^#^	9.22 ± 13.58	1.51 ± 1.07	7.44 ± 10.29
AUC_0-∞_	ng h/mL	90.24 ± 35.81	137.77 ± 93.26	1.14 ± 1.05	506.31 ± 61.62	64.25 ± 20.15^∗^	376.12 ± 50.03^#^	138.89 ± 45.86^#^	14.35 ± 15.92	3.32	10.39 ± 13.73
thalf	h	2.21 ± 1.6	0.9 ± 0.68^∗^	0.12 ± 0.05	5.01 ± 1.68	4.39 ± 0.52	4.44 ± 0.73	4.43 ± 0.89	0.18 ± 0.13	0.21	0.36 ± 0.11
MRT	h	3.92 ± 3.48	1.03 ± 0.41^∗^	0.29 ± 0.05	9.22 ± 0.97	9.18 ± 0.53	7.37 ± 0.61^#^	6.54 ± 1.39^#^	0.44 ± 0.21	0.47	0.66 ± 0.2

*The data are represented as the mean ± SD, *n* = 6. ^∗^*P* < 0.05, comparison between the TE group and HDP group. #*P* < 0.05, CAP group and FAP group compared with TE group*.

#### The Plasma Concentration-Time Profiles and Pharmacokinetic Parameters of TSG

After 5 groups of rats were given different parts of PM, the content of TSG in rat plasma were determined by LC-MS/MS. The contents of the TSG were not detected in PTP group and FAP group of rat plasma due to the low concentration of these two extracts.

The above results showed that the contents of TSG in total part and HDP group rats plasma were higher than those in the other group because of the high concentration of these two extracts. The low TSG plasma concentration of CAP group was caused by only 7.5 mg/kg body weight of the administrated dose. *T* test was used to compare the pharmacokinetic parameters difference between the total group and HDP group. The results showed that the C_max_ of HDP group was significantly higher, and MRT and t_1/2_ were less than the TE group. However, the administrated dosage of TSG in HDP (156.35 mg/kg) is lower than that in the TE group (212.23 mg/kg). This indicated that some components in the TE (with content lower or absent in HDP) could change the pharmacokinetic parameters of TSG in rats by influencing relevant drug metabolic enzymes, such as CYP, UGT, ADH, or others.

#### The Plasma Concentration-Time Profiles and Pharmacokinetic Parameters of Emodin

After 5 groups of rats were given different parts of PM, the contents of emodin in rat plasma were determined by LC-MS/MS. The contents of emodin were not detected in PTP group rat plasma due to the low concentration of the extracts.

The administrated dosages of emodin was 4.91, 0.11, 0.23, and 3.67 mg/kg (body weight) in TE, HDP, CAP, and FAP extracts. The dosage of emodin in CAP group was much lower than that in the total part and FAP groups, but the C_max_ was much higher than those in the plasma after administration. First, the dosage of emodin in FAP group was similar to the TE group, but the C_max_, AUC_0-24_, and AUC_0-∞_ were significantly lower than those in the TE group. Second, the dosage of emodin in CAP group was only 0.23 mg/Kg, but the C_max_, AUC_0-24_, and AUC_0-∞_ were much higher than those in the DCM group. Third, the numerical value of AUC_0-24_ and AUC_0-∞_ were the sum of HDP, CAP, and FAP groups. Therefore, it can be speculated that a portion of emodin was generated through EDG deglycosylation *in vivo*, based on the above points.

#### The Plasma Concentration-Time Profiles of EDG and Pharmacokinetic Parameters

After the 5 groups of rats were given different parts of PM, the contents of EDG in rat plasma were determined by LC-MS/MS. The results of total, HDP, and CAP groups are shown in **Figure 5C** and **Table 7**. According to the dose and concentrations in rat plasma, the EDG in these three groups might have a low bioavailability and fast elimination. The pharmacokinetic behavior of EDG was not compared with each other due to the incomplete plasma concentration data in this study.

The bioavailability of EDG might be low and elimination was fast in rats after administration of these three extracts, and the concentration point data in rat plasma are incomplete. Therefore, the pharmacokinetic behavior of EDG in the different extracts was not evaluated in this study.

### The Results of Liver Enzymes Analysis

The cytochrome P450 enzyme system in liver cells is involved in about 70% the clinical drug metabolism. According to reports, 90% the oxidation reactions of clinical drugs were catalyzed by CYP1A2, CYP2C9, CYP2C19, CYP2E1, CYP3A4, etc. The metabolism of P450 enzyme is one of the main reasons of drug-induced liver injury. The inhibition of the CYP450 enzyme system by the exogenous compound can lead to abnormal metabolism of the drug, resulting in liver damage (Corsini and Bortolini, 2013).

**FIGURE 6:**
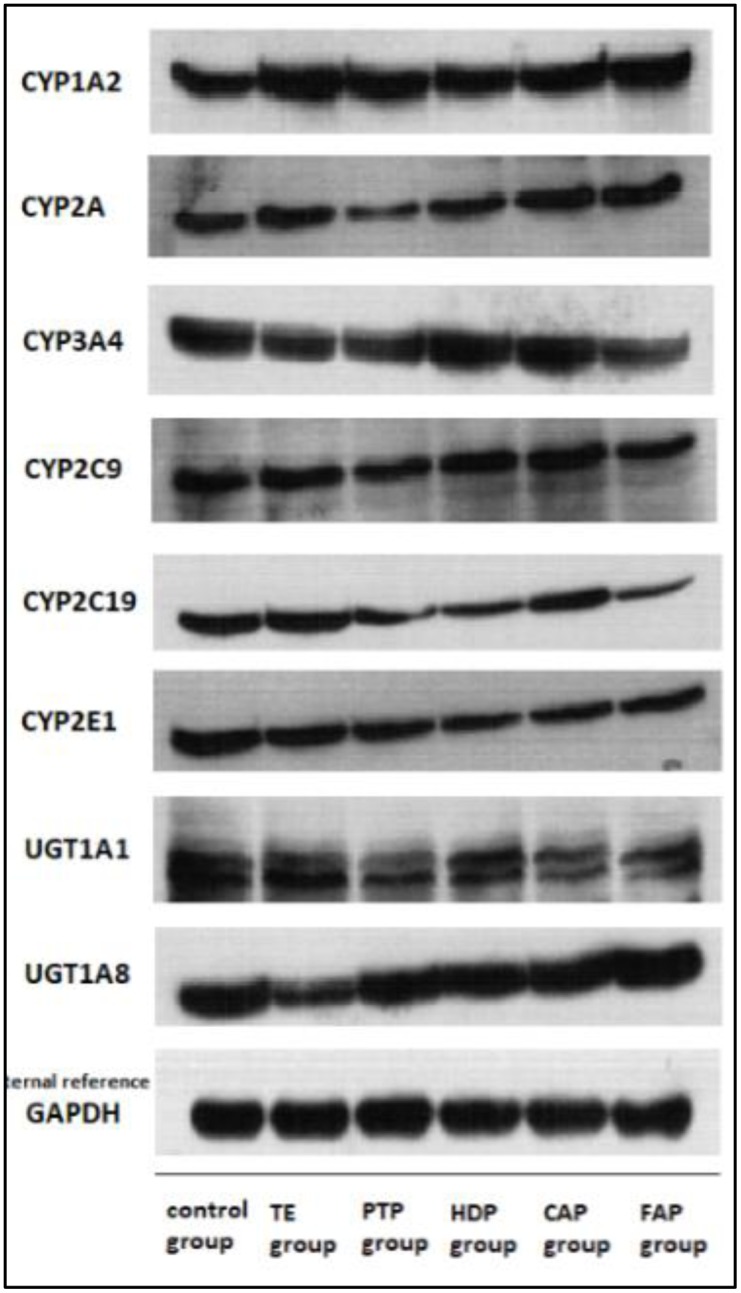
The expression of CYP1A2, CYP2A, CYP3A4, CYP2C9, CYP2C19, CYP2E1, UGT1A1, and UGT1A8 was analyzed by western blot after administration of TE, PTP, HDP, CAP, and FAP.

The WB analysis results of eight hepatic drug enzymes were shown in **Figure 6**, and PM extracts have no significant effect on the expression activity of CYP1A2 and CYP2C9 in the liver of rats. However, parts of the PM extracts produced inhibitory effects on CYP2A, CYP3A4, CYP2C19, CYP2E1, UGT1A1 and UGT1A8. PTP, HDP, CAP, FAP had inhibitory effect on CYP2E1, CYP2E1 not only got involved in drug metabolism, but also catalyzed the activation process of many toxic substances and reduced its activity, increased the levels of this kind of toxic elements in the body or prolonged the residence time, causing liver damage; on the other hand, the decreasing activity of CYP2E1 leaded to the liver injury caused by excessive accumulation of some metabolic waste in the body that were previously metabolized by CYP2E1. CYP3A4 had the highest expression abundance in P450 enzymes, it had a wide range of specificity of substrate, involving in half of the metabolic process of clinical application. The researches have shown that CYP3A4 takes part in a variety of metabolic activation process caused by drug-induced liver injury (Walgren et al., 2005; Zhou et al., 2007). CYP family is mainly involved in electron transfer pathway in the process of oxidative stress, involved in oxidative phosphorylation. The abnormal expression of members of CYP family influences proton gradient, inhibits electron transfer and induces oxidative stress, leading to liver damage (Go et al., 2015; Lin L. et al., 2017). The results of the preliminary proteomics study showed that the hepatic injury induced by PM was associated with the pathway of oxidative phosphorylation, which was consistent with the results of this study. Bilirubin is mainly converted from UGT1A1 enzyme into water-soluble bilirubinic acid conjugate *in vivo*, and then secreted from liver cells and eliminated from bile. The inhibition of UGT1A1 enzyme can make the bilirubin metabolism be metabolic disorders, and induce toxicity (Vitek and Ostrow, 2009; Ma et al., 2013).

## Discussion

The hepatotoxicity studies indicated that the hepatotoxicity of HDP was stronger than other extraction parts *in vivo*, which was consistent with the results *in vitro*. This result also confirms the credibility of the results of previous speculation that tetrahydroxystilbene-*O*-(galloyl)-hex and emodin-*O*-hex-sulfate may be the main toxic ingredient in PM (Lin L.F. et al., 2017). The hepatotoxicity studies showed that liver injury also occurred in others three separated parts administrated groups (PTP, CAP, and FAP), and the serum AST, ALT, and ALP levels of these three groups were significantly higher than those of the control group. The hepatotoxicity of these three groups were different from the results of our previous studies *in vitro.* This indicates that the hepatotoxicity of some components in PM is different between *in vivo* and *in vitro*.

The hepatotoxicity of emodin has prompted detailed instructions in the US National Toxicology Program by a 2-year experiment *in vivo* (National Toxicology Program, 2001). This also indicated that the liver toxicity of group FAP is mainly due to emodin and other free anthraquinone. Emodin was also detected in CAP group rat plasma; the C_max_ and AUC were significantly higher than that of the FAP group, but the dosing of emodin in CAP group was 1/15 of FAP group. Thus, the emodin in CAP group rat plasma might be generated through EDG deglycosylation, which also might be the reason of hepatotoxicity about CAP group *in vivo*. The results of the extract composition analysis based on HPLC and UPLC-Q-TOF/MS showed that PTP is mainly composed of polysaccharides and tannins, and virtually does not have other measurable components, such as EDG, TSG, and emodin (Lin L.F. et al., 2017). Therefore, it is not yet possible to determine the hepatotoxic constituents of this part of extract, and still need further study.

Furthermore, the hepatotoxicity performance of total group was different from other groups in this study. The serum AST, ALT, and ALP levels of TE group were not significantly higher than those of the control group. However, the serum TBil and DBil levels of the TE group were significantly higher than those of the control group, but the other administration group did not have this variation tendency. It is indicated that the type of liver injury in the total group was different from other groups. The reason for these results might be related to the different composition of the five extracts, which were administrated to the five treated groups of rats. The pharmacokinetic behavior of these five extracts ingredients was also different due to the interaction between the components: t_1/2_ and MRT of TSG in TE group was longer than that of the HDP group, but the C_max_ was lower than that of the HDP group even though the dosage (TSG content) was higher. A similar situation was noted with emodin.

The pharmacokinetic studies showed that the C_max_, T_max_, AUC, t_1/2_, and MRT of the major compounds (TSG, EDG, and emodin) of different extracted parts were significantly different in rat plasma. This also indicated that the different components composition could change the pharmacokinetic parameters of compounds in rats by influencing relevant drug metabolic enzymes. PM extracts produced inhibitory effects on CYP2A, CYP3A4, CYP2C19, CYP2E1, UGT1A1 and UGT1A8. The inhibition of the CYP450 enzyme system and UDP-glucuronosyltransferase by the exogenous compound can lead to abnormal metabolism of the drug and bilirubin, resulting in liver damage. Further, it would further affect the liver toxicity effect of PM.

## Conclusion

PM is a widely used herbal medicine in many patented drugs and prescriptions, and the hepatotoxicity induced by PM has received significant attention. This study interpreted the hepatotoxicity effects of PM through comparing the pharmacokinetic behavior of the main components (TSG, EDG, and emodin) of different extraction parts of PM. Liver injury occurred in all the administrated groups, but the hepatotoxicity performance of the total group is different from that of the other groups. The pharmacokinetic behavior of TSG, EDG, and emodin of these five extracts was also different from each other. The pharmacokinetic results showed that the C_max_, T_max_, AUC, t_1/2_, and MRT of TSG, EDG, and emodin of the different extracted parts were significantly different in rat plasma (emodin might be the hepatotoxicity components in PM; Lin et al., 2015a). These results indicated that emodin-*O*-hex-sulfate, tetrahydroxystilbene-*O*-(galloyl)-hex, emodin (original and generated through EDG deglycosylation), and other free anthraquinone might be the reason for the hepatotoxicity of PM *in vivo*. It also indicated that there might be an interaction between the different components composition, which might influence relevant drug metabolic enzymes, such as CYP, UGT, ADH, or others. Stilbene glucoside could inhibit the glucuronidation of emodin in rats through the down-regulation of UGT (Ma et al., 2013). The expression of liver drug enzymes by PM extracts showed that PM extracts have effect on the expression of CYP3A4, CYP2C19, CYP2E1, UGT1A1 etc., And these effects may be related to its hepatotoxicity, but still need further study. Such pharmacokinetic comparisons and interactions would offer useful guidance for understanding the toxicological effects of PM.

## Author Contributions

MZ and LL conducted the main experiments and wrote the manuscript. HL prepared the figures. LY and CQ determined the biochemical and antioxidant indexes. JN conceived the study.

## Conflict of Interest Statement

Partial results of some hepatotoxicity experiments have been published in the article “Application of iTRAQ-Based Quantitative Proteomics Approach to Identify Deregulated Proteins Associated with Liver Toxicity Induced by *Polygonum multiflorum* in Rats. Cell Physiol. Biochem. 2017; 43:2102–2116”. This article was focuses on proteomics, but the manuscript submitted to “Frontiers in Pharmacology” was focuses on the relationship between hepatotoxicity and pharmacokinetics. The content of the article is very different.
